# Socioeconomic impact of chronic delta hepatitis in Spain: Indirect costs of work absenteeism, presenteeism, and premature mortality

**DOI:** 10.1371/journal.pone.0324834

**Published:** 2025-06-20

**Authors:** María Buti, Joaquín Cabezas, Raquel Domínguez-Hernández, Helena Cantero, Miguel Ángel Casado

**Affiliations:** 1 Hospital Universitari Vall d’Hebron and CIBEREHD del Instituto Carlos III, Barcelona, Spain; 2 Servicio de Gastroenterología y Hepatología. Grupo de Investigación Clínica y Traslacional en Enfermedades Digestivas. Instituto de Investigación Valdecilla (IDIVAL). Hospital Universitario Marqués de Valdecilla. Santander, España, Spain; 3 Pharmacoeconomics & Outcomes Research Iberia (PORIB), Madrid, Spain; 4 Gilead Sciences, Madrid, Spain; Al-Azhar University, EGYPT

## Abstract

**Introduction:**

Patients with chronic delta hepatitis (CDH) exhibit higher levels of morbimortality than those with hepatitis B only, generating higher indirect costs for society. The aim of this study was to estimate the loss of productivity and costs resulting from work absenteeism and presenteeism as well as premature mortality among patients with CDH in Spain.

**Methods:**

Patients with CDH in their working age (between 20–65 years) were estimated by an epidemiological flow model that incorporated the prevalence of infection with the hepatitis B and D viruses. To calculate the costs (year-2023) of absenteeism and presenteeism (over a time horizon of 1 year) as well as premature mortality (i.e., the period from death to expected retirement age), as measured in years of productive life lost (YPLL), the human capital method was used. Specific variables pertaining to the Spanish labour market (working population with hepatitis D virus (HDV), working days, average number of hours worked and gross annual salary) were considered for each sex and age group and distinguished based on infection status. All parameters were obtained from the literature and Spanish databases.

**Results:**

A total of 1,313 CDH patients of working ages (59% men, 41% women) and 97 patients who performed unpaid housework were identified. A total of 300,113 working hours were lost per year (29,015 hours/absenteeism and 271,098 hours/presenteeism), which entailed total annual costs of €4.5M (€536,400/absenteeism and €3.9M/presenteeism) related to CDH. Among patients of working ages, CDH was estimated to cause 28 annual deaths at a cost of €8.2 M, resulting in 449 YPLL at an average cost of €18,297/YPLL. The indirect costs were estimated to be € 12.7M.

**Conclusions:**

CDH entails significant economic burdens for society. Consideration of the indirect costs associated with CDH is crucial with respect to the design of public health policies and interventions.

## Introduction

Approximately 5% of patients with chronic hepatitis B worldwide are coinfected with HDV [1]. In Spain, although epidemiological data are scarce, the prevalence of hepatitis B surface antigen (HBsAg) is estimated to be 0.22% [2]; among HBsAg positive patients, 7.7% are infected with HDV [2–4].

Chronic delta hepatitis (CDH) is one of the most severe types of viral chronic hepatitis. HBV/HDV coinfection is associated with a more rapid progression of liver disease, thus increasing the risk of liver complications such as liver decompensation or hepatocellular carcinoma or even death [5,6]. Furthermore, despite recommendations regarding screening in all HBsAg positive for hepatitis D, many patients remain undiagnosed and untreated [7]. In most cases, unawareness of the infection involves a delayed the diagnosis that is then made in more advanced stages of liver disease, thus leading to patient’s health worsening [8]; this situation entails various challenges for health systems.

In view of the greater morbidity associated with CDH, this type of hepatitis is associated with decreased quality of life and health-related activity [9]. The daily activity of patients with CDH worsens and can affect their work capacity by decreasing their productivity, potentially even leading to disability. Furthermore, coinfected patients are younger and exhibit lower survival rates [10], thus leading to premature mortality.

Similarly, these effects on the health of CDH patients represent economic burdens for health systems and society as a whole [8,11]. The development of liver complications, in addition to the high health costs related to medical care that they entail, results in social costs related to the loss of productivity [11] or premature mortality among patients of working age [12,13].

In the context of decision-making, when the social impact of a disease is high, in addition to the health costs derived from health care, one must consider the impact of the disease on society. Some studies have analysed the costs of hepatitis C or B from a social perspective [14–16], but none hepatitis D. Accordingly, the objective of this analysis was to estimate the loss of productivity and costs associated with absenteeism and occupational presenteeism as well as premature mortality among patients with CDH in Spain.

## Methods

**Fig 1:**
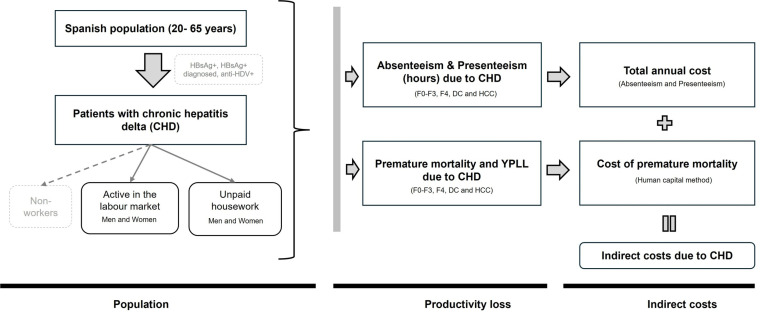
Scheme used for the cost model. Anti-HDV: antibody to hepatitis D virus; HDV-RNA: hepatitis D virus ribonucleic acid; *YPLL, years of productive life lost*; CC: compensated cirrhosis; DC: decompensated cirrhosis; HCC: hepatocellular carcinoma; F: grade of fibrosis; CD: chronic delta hepatitis; HBsAg: hepatitis B virus surface antigen; HDV: hepatitis D virus; HCD: chronic delta hepatitis; HDV: hepatitis B virus surface antigen; HDV: hepatitis B virus; HCV: hepatitis B virus; HC: compensated cirrhosis; DC: decompensated cirrhosis; HCC: hepatocellular carcinoma; F: grade of fibrosis; HDV: hepatitis B virus surface antigen.

For the calculation of indirect costs, the analysis takes into account the population infected with chronic hepatitis D and the loss of productivity (Fig 1).

**Fig 2:**
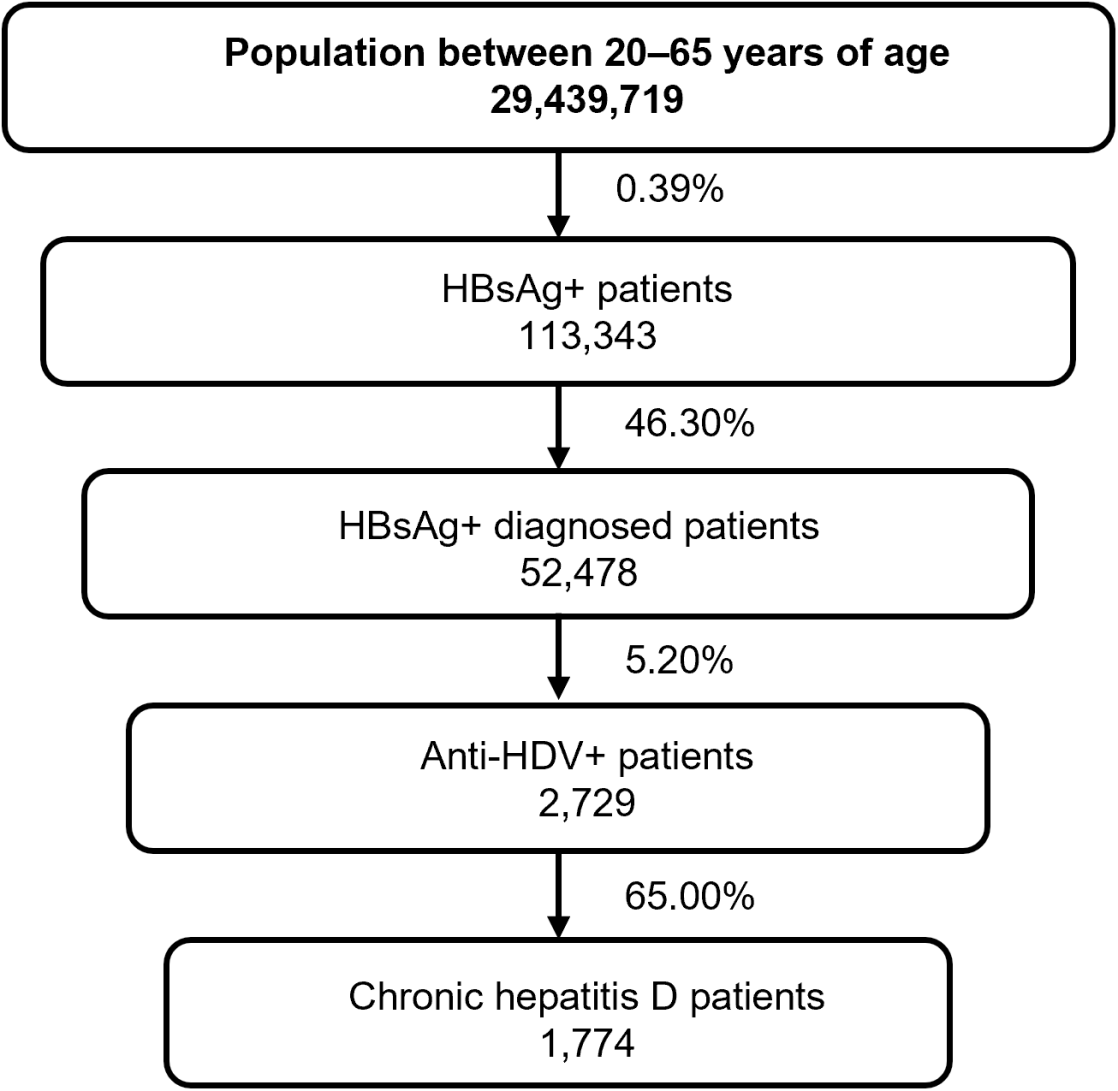
Hepatitis D patient flow. Anti-HDV: Hepatitis D virus antibody, HDV-RNA: Hepatitis D virus ribonucleic acid, HBsAg: Hepatitis B virus surface antigen, CDH: Delta chronic hepatitis.

The total number of CDH patients of working age was calculated on the basis of an epidemiological flow model considering the Spanish population between 20 and 65 years old [17], the prevalence of HBsAg [2,4,18] and the percentage of patients who had received such a diagnosis [19], the seroprevalence of anti-HDV [4], and the proportion of patients with detectable HDV-RNA [3]. All of these data were obtained from previous published data that are available in the literature (Fig 2).

The analysis conducted for this research took the percentage of men and women into account [18] across various age ranges [9]. The percentages of patients with CDH employed and of people engaged in unpaid housework were obtained from a study that investigated the Spanish population [9]. The infected population of working age that works was subsequently distributed proportionally among the different age ranges according to sex [20]. All data associated with this analysis were validated by a panel of experts.

The analysis was conducted from a social perspective. Loss of productivity was measured in terms of both absenteeism defined as the time at work lost due to absence from work within the legal working day, and presenteeism, the time in which a reduction in productivity occurred during the legal working day as a result of the presence of a disease over a time horizon of one year, as well as in terms of the premature mortality associated with the disease, i.e., the period from death to expected retirement age [21].

The human capital method was used to translate the loss of productivity into economic terms. As part of this method, the indirect costs are quantified considering the reduction in the future gross earnings of patients due to the morbidity or mortality caused by the disease, which are determined by the wages. Through this method, a worker’s wage gain can be used to estimate the labour productivity lost due to illness [21,22].

The percentages of absenteeism and presenteeism were obtained from a study that aimed to collect information regarding the quality of life of patients with CDH, including the associated morbidity, and to describe the deterioration of labour productivity using the work productivity and activity impairment questionnaire. Values were given for patients in different states of fibrosis or hepatocellular carcinoma. No values were given for patients with decompensated cirrhosis due to the lack of patients with paid employment in this stage of health in that study [23]. The deterioration in productivity was assumed to remain constant throughout the entire time horizon of the analysis. These percentages were used to calculate the total number of annual work hours lost per patient.

To calculate the number of working days per year, a working week of five days was assumed over 52 weeks, minus 30 days of vacation, compulsory leave and holidays [24]. The cost per hour of work was estimated on the basis of the average annual gross salary of a person in Spain (year 2023) by age range and sex [25,26] as well as the average number of annual hours worked by sex and age, given average weekly working hours (39.9 hours for men, 35.3 hours for women and 22.6 hours of housework) [25,27] (Table 1).

**Table 1:** Parameters used to conduct the analysis.

	Value	References
Annual number of working days	243	[24]
Average number of hours worked per week		
*Men*	39.9 hours	[20]
*Women*	35.3 hours	[20]
*Unpaid housework*	22.6 hours	[27]
Average labour costs per hour		
*Men*	16.6-23.0€	[25–26]
*Women*	13.0-20.4€	[25–26]
*Unpaid housework*	€8.5	[26]

Premature mortality was estimated from mortality resulting from CDH (6.7%) [28] and the time from the patient’s diagnosis to death (measured in years) by health status [29] as well as by age range [9]. In addition, the years of productive life lost (YPLL) were estimated considering the retirement age of the Spanish population minus the age at which the patient died due to CDH.

### Sensitivity analysis

Different univariate sensitivity analyses were conducted modifying the rate of HBsAg positive (0.2–0.6%) and rate of HBsAg positive patients diagnosed (34.9–58.1%), assuming a loss of productivity in patients with decompensated cirrhosis similar to that associated with cirrhotic patients as well as varying the salary income by ±10% of the salary.

## Results

A total of 1,774 working-age CDH patients were estimated; among these patients, 1,313 were active (59% men, 41% women), while 97 patients were engaged in unpaid housework.

The total amount of time lost due to absenteeism was 29,015 hours, and the corresponding figure concerning presenteeism was 271,098 hours, accounting for a total of 300,113 annual work hours lost due to CDH.

**Fig 3:**
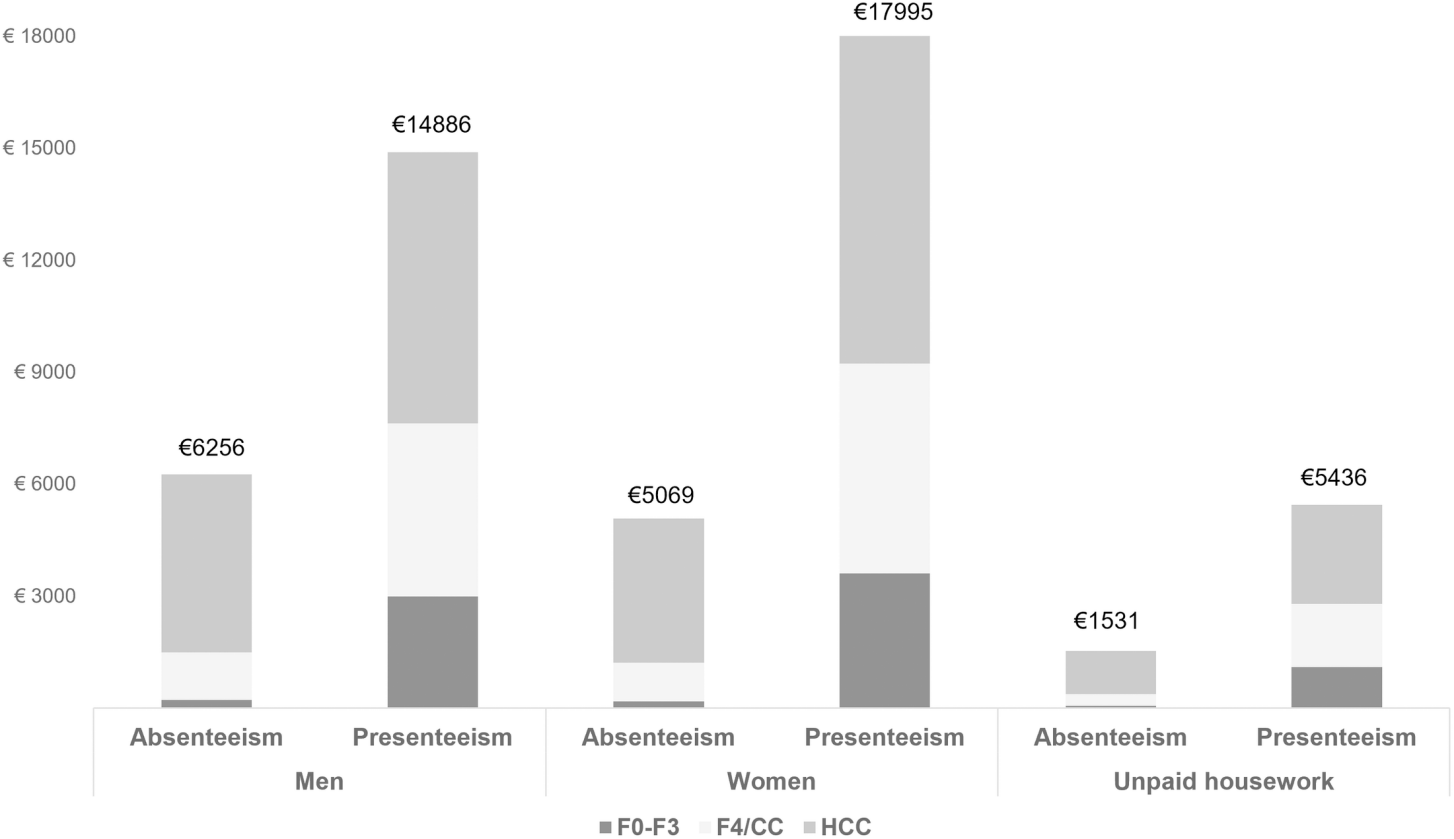
Total costs per patient pertaining to absenteeism and presenteeism according to health status as well among working men, working women and individuals performing unpaid housework * CC: compensated cirrhosis; HCC: hepatocellular carcinoma; F: degree of fibrosis.

The annual costs for absenteeism were € 536,400, and the corresponding figure for presenteeism was € 3.9 million, resulting in total annual costs of € 4.5 million related to CDH. The total costs per patient according to health status as well as the corresponding costs among working men, working women and people performing unpaid housework are presented in Fig 3.

CDH caused 28 annual deaths (22 in the labour force and 6 among individuals engaged in unpaid housework), resulting in a total of 449 YPLL (Table 2). The costs attributable to premature mortality were € 8.2 million, for an average of € 18,297 per YPLL (€ 19,649 among women, € 23,849 among men, and € 6,629 among individuals performing housework).

**Table 2:** Number of premature deaths and YPLL related to premature mortality according to fibrosis status, sex and unpaid household chores as well as the corresponding costs.

Health states	Women (workes)			Men (workers)			Unpaid housework		
	*Premature deaths due to HDV*	YPLL	*Total cost*	*Premature deaths due to HDV*	YPLL	*Total cost*	*Premature deaths due to HDV*	YPLL	*Total*
F0-F3	6	70	€1,331,589	8	100	€2,316,186	4	58	€382,110
F4 CC	1	22	€442,684	2	32	€775,210	1	17	€114,778
F4 CD	2	41	€828,979	3	59	€1,454,481	1	31	€208,633
CHC	<1	6	€116,985	<1	8	€205,295	<1	4	€29,354
**Total**	**9**	**138**	€**2,720,237**	**13**	**199**	€**4,751,172**	**6**	**111**	€**734,875**

*YPLL, years of productive life lost; CC, compensated cirrhosis; DC, decompensated cirrhosis; HCC, hepatocellular carcinoma; F, fibrosis grade.*

* Nonactive people engaged in unpaid housework

**Fig 4:**
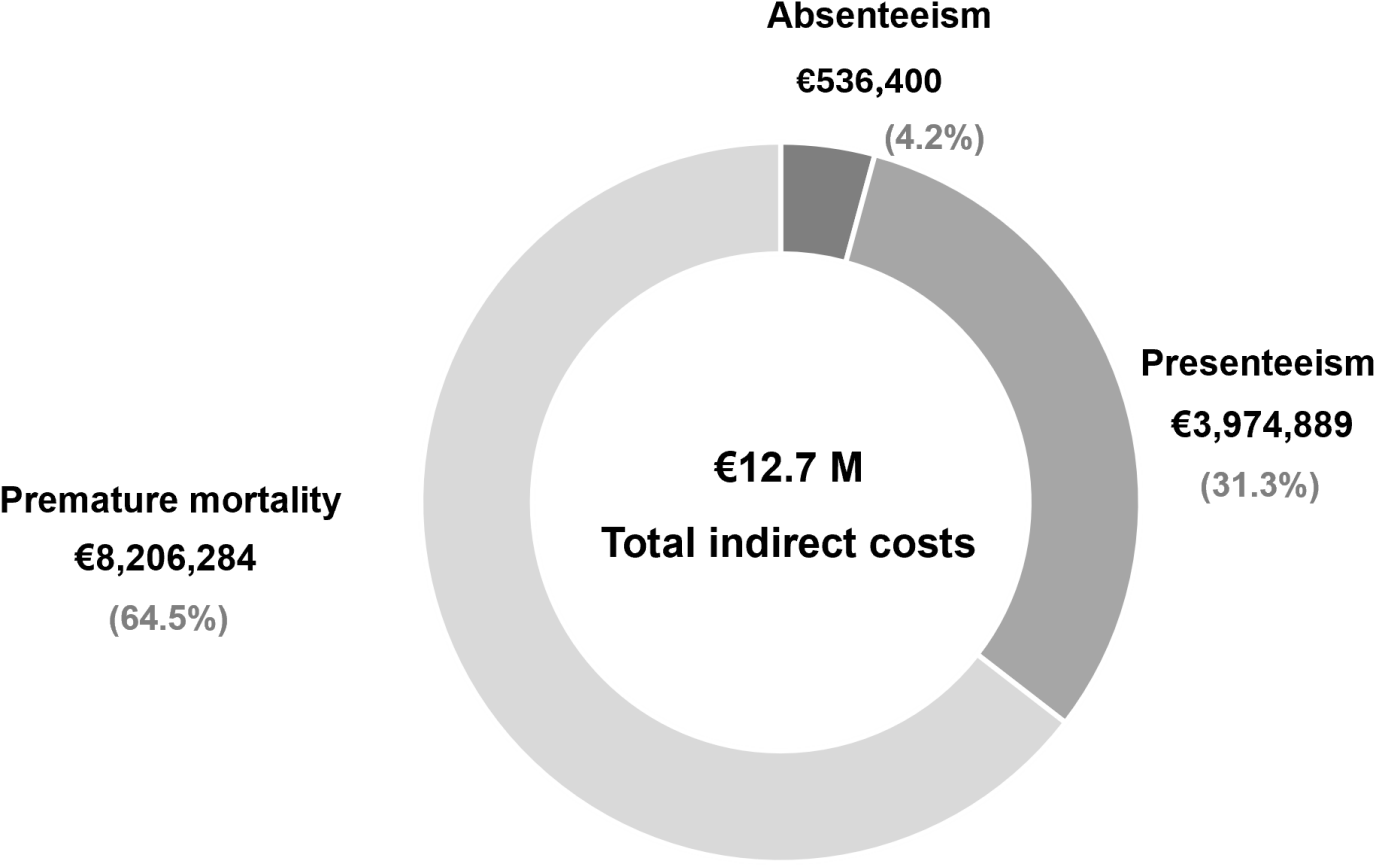
Total costs pertaining to absenteeism, presenteeism and premature mortality among patients with chronic delta hepatitis.

The total indirect costs related to the loss of productivity resulting from absenteeism, presenteeism and premature mortality reached € 12.7 million per year (Fig 4).

The sensitivity analyses revealed variation in the total indirect costs, which ranged between € 6.6M and € 19.8M; the most influential parameter was the changes in the prevalence of HBsAg positive. The consideration of absenteeism and presenteeism among patients with decompensated cirrhosis led to variations in the total indirect costs between € 13.0M and € 14.0M. The variation in the wage costs entailed that the total indirect costs varied between € 12.0-13.4M for working men, € 12.2-13.2M for working women, and € 12.6- € 12.8M for patients performing unpaid housework (Table 3).

**Table 3:** Results of the sensitivity analysis.

Sensitivity analysis	Sensitivity analysis scenarios	Original value	Total costs			Total cost
			Absenteeism	Presenteeism	Premature mortality	
		**Baseline case**	***€536,400***	***€3,974,889***	***€8,206,284***	***€12,717,573***
SA1	HBsAg+ patients (0.2%)	*0.385%*	€278,649	€2,064,877	€4,263,005	€6,606,531
SA2	HBsAg+ patients (0.6%)		€835,947	€6,194,632	€12,789,015	€19,819,594
SA3	HBsAg+ diagnosed patients (34.9%)	*46.3%*	€403,748	€2,991,898	€6,176,869	€9,572,514
SA4	HBsAg+ diagnosed patients (58.1%)		€672,874	€4,986,211	€10,294,190	€15,953,275
SA5	Loss of productivity resulting from absenteeism among CD patients (4.7)	*0*	€846,840	€3,974,889	€8,206,284	€13,028,014
SA6	Loss of productivity resulting from presenteeism among CD patients (25.6)	*0*	€536,400	€5,315,951	€8,206,284	€14,058,635
SA7	Wage costs (men) (±10%)	*----*	€502,742 - €570,058	€3,764,102 - €4,185,676	€7,731,167 - €8,681,402	€11,998,010 - €13,437,136
SA8	Wage costs (women) (±10%)	*----*	€517,448 - €555,351	€3,797,814 - €4,151,964	€7,934,261 - €8,478,308	€12,249,522 - €13,185,624
SA9	Wage costs (*housework*) (±10%)	*----*	€535,369 - €537,430	€3,965,263- €3,984,515	€8,132,797 - €8,279,772	€12,633,429 -€12,801,717

HBsAg: hepatitis B surface antigen, HDV-RNA: hepatitis D virus ribonucleic acid, CD: decompensated cirrhosis, HCC: hepatocellular carcinoma, HDV: hepatitis D virus.

## Discussion

Economic evaluations are useful tools in the context of decision-making with regard to the prioritization of health interventions that can improve people’s health efficiently. For this purpose, it is necessary to consider not only the direct health costs, as has been done in most cases, also the indirect costs related to the loss of productivity.

In light of the severity of CDH and its rapid progression, delayed or absent diagnosis allows advanced liver disease and decompensation to emerge, thus harming the health of people with CDH and affects their daily and work activities CDH [9]. This analysis explores this impact in economic terms by estimating losses in labour productivity. And highlight the importance to increase early diagnosis by performing automatic reflex testing to all HBsAg positive patients, RNA testing to all HDV antibody and linking to care for disease management to all HDV patients.

In the context of progressive chronic diseases, it is essential to assess patients’ ability to perform their usual tasks and fulfil their responsibilities during their working days. In situations involving, patients with advanced liver disease and liver complications demonstrate a greater loss of productivity than do those in milder stages. Therefore, in line with the course of the disease, this analysis explored absenteeism and presenteeism on the basis of the different health states exhibited by patients. The results revealed that patients with hepatocellular carcinoma were associated with higher indirect costs than were patients in milder stages of the disease. Namely, patients with hepatocellular carcinoma exhibit poorer quality of life and are absent from work more frequently with respect to HBV monoinfected.

Our analysis also evaluated the loss of productivity resulting from premature mortality. The results showed that due to the severity of the disease and the fact that patients with HDV are relatively young, many years of life are lost, and high costs are generated among patients in milder stages of the disease.

On the other hand, although average wages were used in the analysis to estimate productivity loss, in consideration of prospective wage fluctuations given their variability across sectors or regions, sensitivity analyses were conducted with regard to increasing wages. These analyses revealed that the economic burden could reach important economic levels for society, i.e., between € 6.6 M and € 19.8 M. To our knowledge, no studies on the indirect costs of chronic hepatitis D in Spain have been published up to now. However, if we take into account the severity of this disease with respect to other types of viral hepatitis, our results are in line with those of other analyses that have evaluated these same costs in other hepatitis-related settings [12,13,15]. In addition, our results, which highlight the differences corresponding to sex and different health states on the part of the patient, are disaggregated, thus making direct comparison difficult.

Our study has certain limitations. Absenteeism and presenteeism values were not available for patients with decompensated cirrhosis. Namely, the study from which these values were drawn did not report any results regarding these patients because at that time, none of them were active at work. To ensure that the findings of this research remained conservative, the base case of our analysis was conducted without taking into account the indirect costs associated with these patients due to lost time or the occurrence of disability during work. Some studies have reported that the progression of the disease, as in the case of decompensated cirrhosis, decreases patients’ quality of life, thus affecting their labour productivity [9]. Therefore, the lack of direct data may underestimate the true economic burden of CDH in advanced stages. Accordingly, a sensitivity analysis was conducted that assumed a value similar to that associated with patients with compensated cirrhosis. The results of this analysis revealed an increase of nearly 10% in presenteeism with respect to the total indirect cost of the base case. Another limitation pertains to the time horizon of this analysis. At present, little evidence is available concerning how CDH affects the work activity of patients. For this reason, this analysis was conducted on the basis of a time horizon of one year in terms of absenteeism and presenteeism. However, in consideration of the chronic nature of CDH, a longer time horizon may show a more complete picture of the economic consequences of the disease, especially considering the progressive nature of liver disease. Future studies should monitor patients in terms of their absence from work, moreover, how CHD disease progression could further impact absenteeism/presenteeism in the longer run. In addition, although recommendations on economic evaluations consider including ethical or equity aspects, which could affect the results, it is difficult to capture the full complexity of the labour market in modelling. However, wage differentials between men and women have been considered to take into account the gender perspective in the analysis. Finally, although the analysis is specific to Spain and therefor the results may not be generalisable to other countries with different health systems, labour markets or epidemiological profiles of CDH, it shows the economic impact of the disease related to social costs.

Diagnosis and early treatment for viral hepatitis are associated with clinical benefits by preventing progression of liver disease to cirrhosis and decompensation as well as HCC. In the context of chronic HDV infection, there is a paucity of scientific evidence; but it can be expected similar outcomes than those reported in in patients with chronic hepatitis B and C [8,30,31]. The implementation of the reflex test for hepatitis D among HBsAg-positive people allowing an increase in the detection and diagnosis of HDV infection, has been associated with a reduction of the clinical and economic burdens associated with HDV infection, as has recently been demonstrated [8]. This finding highlights the need to promote the performance of anti-HDV antibody determination with the aim of preventing delays in diagnosis. An improvement in patients’ prognoses leads to improved states of health and can mitigate losses in terms of the number of hours worked.

Patients with hepatitis B and/or hepatitis C who achieve viral suppression or HCV eradication have an improvement in quality of life in comparison with viremic patients. [16,32]. Moreover, utility also declines with disease progression from non-cirrhosis to cirrhosis or decompensation [31]. At present, only 10% of patients are treated with interferon [33], this treatment is associated with low levels of effectiveness and high levels of adverse effects, thereby interfering with the quality of life of patients [34]. The introduction of bulevirtide, a drug that is associated with better efficacy and an improved safety profile [33], has led to improvements in patient-reported outcomes (PROs), as well as avoiding liver complications [30]. In other cases of chronic viral hepatitis, such improvement in PROs has positive effects on the social and economic burdens resulting from the loss of productivity [35,36]. Consequently, future studies should compare the impact of bulevirtide on quality of life with that of current regimens and explore the ways in which this treatment influences social costs.

## Conclusions

CDH is associated with a significant economic burden on society due to the loss of productivity and premature mortality. Consideration of the indirect costs associated with CDH is crucial for the design of public health policies and interventions.
